# Remote Monitoring of Psoriasis: Comparing Care Models and Evaluating Quality of Life Outcomes: Mixed Methods Study

**DOI:** 10.2196/73664

**Published:** 2025-06-03

**Authors:** Jana Arsenjeva, Priit Kruus, Riina Hallik, Secil Matasova, Laura Prett, Katrin Kaarna, Liisi Raam, Oliver Taul, Liis Ilves, Kaisa Viljar, Pille Konno, Peeter Ross, Külli Kingo

**Affiliations:** 1 Tallinn University of Technology Tallinn Estonia; 2 Dermtest OÜ Tallinn Estonia; 3 Linnamõisa Family Medicine Centre Tallinn Estonia; 4 University of Tartu Tartu Estonia; 5 Tartu University Clinic, University of Tartu Tartu Estonia; 6 Confido Healthcare Centre Tallinn Estonia; 7 East Tallinn Central Hospital Tallinn Estonia

**Keywords:** psoriasis, remote management, primary care, specialist care, collaborative care, teledermatology

## Abstract

**Background:**

Remote monitoring is increasingly used in psoriasis care, with the International Psoriasis Council endorsing teledermatology (TD) as a feasible alternative to in-person visits. While evidence supports specialist-led remote monitoring, limited research exists on its effectiveness in primary care. A publicly reimbursed remote monitoring program was piloted at both primary and specialist care levels, enabling comparative analysis of quality of life outcomes and offering insights into factors contributing to better management outcomes.

**Objective:**

This study aims to evaluate the feasibility and effectiveness of remote psoriasis monitoring at both primary and specialist care levels, and to identify factors associated with improved outcomes in primary care.

**Methods:**

This study used a retrospective convergent parallel mixed methods approach to analyze data from 110 patients enrolled in a publicly reimbursed remote monitoring program between January 2022 and July 2023, across 7 family medicine centers and 1 outpatient dermatovenerology center. Data sources were public insurance claims and a TD platform, which captured the Dermatology Life Quality Index (DLQI) and related metrics. The effectiveness of remote monitoring at both care levels was assessed by comparing baseline and average DLQI scores; a minimal clinically important difference (MCID) was defined as ≤–3. Odds ratios were calculated to compare outcomes between care levels. Correlation analysis examined associations between DLQI changes and variables such as baseline Psoriasis Area and Severity Index, demographics, and use of e-consultations. Qualitative insights from patient feedback and system usability surveys were analyzed to illustrate variability in care processes.

**Results:**

The specialist care group (n=39) showed a significant mean reduction in DLQI of –1.33 (P=.01), whereas the primary care group (n=37) demonstrated a nonsignificant change of –0.34 (P=.36). Patients receiving specialist care were 3.91 times more likely to achieve a clinically significant improvement (MCID≤–3) compared with those in primary care (P=.04). Collaborative care between primary care providers (PCPs) and specialists was associated with more favorable changes in DLQI scores at the primary care level (r=–0.339, P=.04). Case series analysis of patients achieving MCID in primary care revealed variability in management approaches, including PCP-led models incorporating collaborative care elements and nurse-led models without such collaboration.

**Conclusions:**

This study is the first to compare remote psoriasis monitoring across specialist and primary care levels. While remote management proved effective in specialist care, outcomes in primary care were nonsignificant, reflecting patterns seen in in-person PCP-led care. Using public claims data, the study highlights that integrating specialist support into PCP-led care via standardized e-consultations may enhance patient outcomes. It underscores the need for further research and the development of standardized collaborative care models between PCPs and specialists in the remote management of psoriasis.

## Introduction

Psoriasis is a chronic inflammatory skin condition affecting approximately 2% of the global population [1-4]. The disease presents a significant burden, impacting physical, social, and psychological well-being, with severity closely linked to reduced quality of life (QoL) [3,5-7]. Psoriasis is also associated with comorbidities such as psoriatic arthritis, cardiovascular disease, and depression, further compounding its burden [5,7,8]. QoL is widely recognized as an essential measure in psoriasis management, reflecting the disease’s broad impact [3,5].

There is a growing shift from traditional in-person specialist consultations to patient-centered remote care models, particularly for chronic dermatologic conditions such as psoriasis [6,9-11]. Teledermatology (TD) has emerged as a cost-effective [12,13] and comparably effective [9] alternative to conventional care, improving accessibility and supporting early intervention [6,7,14]. TD encompasses several approaches, including synchronous (real-time video consultations) [1] and asynchronous store-and-forward systems, which allow clinicians to review patient data remotely at a later time [9,15]. Advanced TD models, such as Collaborative Connected Health, facilitate continuous monitoring through the integration of patient-reported outcomes, image uploads, and asynchronous communication, supporting proactive and timely intervention [8,9]. Remote monitoring is a key component of TD, enabling continuous assessment of disease severity and treatment response beyond traditional clinic visits [6,11].

Collaborative Connected Health models can empower primary care providers (PCPs) to contribute to psoriasis management. Digital platforms equip PCPs with tools to manage patients and submit data for specialist evaluation when needed. This allows specialists to monitor changes in psoriasis severity, adjust treatment plans in a timely manner, and reduce the need for in-person referrals [11]. E-consultations, a form of asynchronous TD, enable PCPs to obtain timely specialist input without requiring real-time interaction [15]. This approach has been shown to improve access to specialists, reduce unnecessary referrals, and streamline decision-making in primary care [16]. Furthermore, collaborative e-consultation models, when integrated with remote monitoring, can enhance continuity of care and optimize treatment adjustments [8,11,14].

The International Psoriasis Council framework outlines the potential role of TD in psoriasis management, emphasizing its capacity to improve accessibility, streamline workflows, and enhance treatment efficiency [17]. While TD offers a viable alternative to in-person visits, its successful integration into primary care remains challenging. Key barriers are limited dermatology expertise, resource constraints, and the need for standardized implementation protocols [18-20].

However, despite growing adoption, there remains limited knowledge about how TD and remote monitoring are integrated into everyday primary care workflows, particularly in models without direct specialist involvement [2,21]. Most existing studies have focused on specialist-led interventions, leaving a gap in understanding the feasibility, effectiveness, and challenges of remote psoriasis care managed by PCPs [6,22,23]. Questions remain regarding whether primary care models can achieve comparable patient outcomes, how remote tools influence clinical decision-making, and how collaborative practices—such as e-consultations—affect care quality. Despite the growing adoption of TD, research on its impact at the primary care level remains limited [18]. PCPs often serve as the first point of contact for patients with psoriasis, yet they face significant challenges, including limited dermatological training [19,24], the complexities of managing multimorbidity [25], and delayed access to specialists [20]. These barriers can result in delayed diagnosis, suboptimal treatment decisions, and insufficient consideration of QoL outcomes [18,19].

Specifically, there is limited evidence on how remote care models influence diagnostic confidence, treatment decision-making, and QoL outcomes in primary care settings [4,9,26,27]. Additionally, implementation barriers, such as limited dermatology-specific training for PCPs, unstructured e-consultation pathways, and inconsistent referral protocols, have not been systematically studied [22,28].

Remote monitoring offers a potential solution by bridging the gap between primary and specialist care, facilitating continuous disease assessment, and supporting early intervention. However, its impact on primary care workflows and patient outcomes remains underexplored.

To address both outcome effectiveness and contextual implementation, this study used a mixed methods approach. Given the variability in care delivery across settings—and the influence of both clinical and contextual factors on outcomes—a combination of quantitative and qualitative methods was essential. The quantitative analysis enabled comparison of QoL outcomes across care levels, while qualitative case series insights provided a deeper understanding of care process variability and collaboration practices in real-world settings. This combination was critical to capturing both measurable impacts and the underlying care dynamics influencing those outcomes—particularly in primary care, where workflows and resources vary widely [29-31].

This study aims to evaluate the feasibility and effectiveness of remote psoriasis monitoring in both specialist-led and primary care settings, identify factors associated with improved outcomes at the primary care level, and examine how collaborative digital tools, such as e-consultations and continuous monitoring systems, affect QoL.

## Methods

### Study Design

The study draws on data from a publicly reimbursed pilot program, implemented as part of a quasi-experimental, multisite trial in Estonia between January 2022 and July 2023, across 7 family medicine centers and 1 dermatovenerology outpatient clinic. This design enabled the integrated analysis of quantitative and qualitative data, providing a comprehensive understanding of the intervention’s effectiveness, feasibility, and real-world implementation. Mixed methods approaches are widely recognized as suitable for evaluating complex health services, particularly when both outcomes and adoption processes are key concerns [29-31].

To comprehensively evaluate both the clinical outcomes and contextual implementation of remote psoriasis monitoring, the study used a retrospective, convergent parallel mixed methods design. Quantitative data included patient-reported Dermatology Life Quality Index (DLQI) scores, clinician-reported Psoriasis Area and Severity Index (PASI) scores, and claims-based health care utilization data. Quantitative analyses, such as comparative effectiveness and correlation techniques, were conducted to measure changes in QoL and identify clinical or demographic factors influencing these outcomes.

To complement the quantitative findings, a qualitative case series was conducted to explore variations in care pathways and management practices at the primary care level. Although no formal thematic analysis was performed, the qualitative component provided illustrative, patient-level insights based on observational and contextual data. These data were drawn from pseudonymized patient feedback questionnaires (administered online via Google Forms; Google LLC/Alphabet Inc) and system usability assessments. This approach enhanced the interpretation of the findings by capturing care process differences and patterns that quantitative analysis alone could not reveal. It was also used to illustrate individual variability and highlight potential mechanisms of improvement.

As a result of feasibility and ethical constraints, patients were assigned to groups based on their level of care (primary or specialist) and willingness to participate. Randomization was not possible within the scope of this publicly funded pilot intervention.

Data sources were a nationwide public insurance claims database covering all publicly reimbursed patient interactions, visits, and medications during the study period, as well as a secure TD platform database where DLQI and other remote monitoring measures were reported.

While the broader pilot project evaluated multiple metrics (eg, adherence, cost, usability), this study specifically focuses on QoL outcomes (DLQI) and the comparative analysis of care models, using a subset of the intervention group data.

### Outcome Measures

The primary outcome of this paper was the effectiveness of remote psoriasis management, measured by changes in DLQI scores across primary and specialist care settings [32,33]. The DLQI, a validated 10-item questionnaire, assesses the impact of psoriasis on QoL. Scores range from 0 to 30, with higher scores indicating greater impairment [34,35]. This tool is widely used in dermatological studies, enabling comparisons and providing insights into factors influencing outcomes [5,35,36].

Effectiveness was first analyzed for the entire intervention group, then compared between primary and specialist care, and finally evaluated separately to provide detailed insights. PASI scores at baseline were used to categorize psoriasis severity (mild: 1-4, moderate: 5-10, severe: >10) [37], offering clinical context for examining the relationship between disease severity and QoL outcomes.

Correlation analysis was conducted to explore clinical factors associated with changes in DLQI scores in primary care. The odds ratio for achieving a meaningful DLQI improvement (minimal clinically important difference [MCID]≤–3) was calculated, based on prior studies defining MCID ranges in dermatological conditions [38,39]. Specific cases that achieved the MCID in psoriasis management at the primary care level were selected for case series analysis, integrating qualitative and quantitative data such as patient feedback questionnaires, claims data [40,41], and medication prescriptions.

### Study Population

This study analyzed data from 110 patients participating in the remote monitoring group across both care levels in the real-life, publicly reimbursed remote monitoring pilot trial. The cohort consisted of 55 men and 55 women, with 84 (76.3%) aged 50 years or younger and 26 (23.6%) aged over 50 years. Inclusion criteria for remote monitoring were (1) a diagnosis of plaque psoriasis, (2) age between 18 and 75 years, (3) access to a smartphone or computer, and (4) willingness to provide informed consent and commit time to the study. Exclusion criteria were (1) a diagnosis other than plaque psoriasis, (2) a history of alcohol or drug abuse within 6 months before the study, (3) inability to fulfill study requirements, and (4) any other reasons deemed by the investigator or sponsor that rendered the patient ineligible.

### Quantitative Data Collection and Exclusion

Data were collected at both specialist and primary care levels. Patient eligibility was first confirmed, and informed consent was obtained, followed by the initial completion of patient-reported outcome measures [42] and PASI assessments, all securely stored in a specialized TD database developed by a TD company for this use case. The study protocol required patients to regularly complete online questionnaires such as the DLQI, Psoriasis Symptoms and Signs Diary [43], and Early Arthritis for Psoriatic Patients [44], facilitated through a web-based platform with a 2-factor authentication log-in. DLQI questionnaires were scheduled to be sent monthly on the 15th, allowing patients to complete them at home. Email reminders, including follow-up notifications, were also sent to encourage timely completion. Doctors were also encouraged to actively engage with patients. The platform enabled real-time transmission of patient-generated data, allowing physicians to securely and conveniently access and review the results. Both PCPs and specialist dermatologists could review this information asynchronously to provide recommendations, initiate treatment adjustments, or refer patients to other specialists as needed.

The System Usability Scale (SUS) was used to assess system usability through a 10-item questionnaire. Participants rated each item on a 5-point Likert scale ranging from 0 (strongly disagree) to 4 (strongly agree). Individual responses were converted and summed, then scaled by a factor of 2.5 to produce scores ranging from 0 to 100, where higher scores indicate better usability [45]. SUS scores were obtained directly from the pilot study without adjustments and were analyzed as part of the case series outcomes in this study.

Additionally, e-consultations were facilitated through the health information system. PCPs submitted detailed referrals to a chosen specialist, including information about the patient’s condition, test results, clinical observations, and secure access to previous remote monitoring data collected at the primary care level. Specialists reviewed the referral and provided recommendations or requested in-person visits, responding within 4 working days. Both the PCP and the patient had access to the consultation records, fostering transparency and efficient communication [46].

The intervention was financed through a public reimbursement model implemented as part of a nationwide remote care pilot project by the Estonian Health Insurance Fund, the sole public payer in the solidarity health care system. This ensured that TD services were accessible to the population without additional costs to the patient [47]. The payment model incorporated value-based elements, including outcome-based payments to participating health care providers at the end of the 1-year remote monitoring period.

Participants were enrolled at different times throughout the study period. To account for this, the first DLQI entry within 45 days of enrollment was considered the baseline score. If the QoL questionnaire was completed twice during the initial or any follow-up month, the average of the 2 scores was used. Each participant’s follow-up timeline was anchored to their individual start date, with the first month of follow-up defined according to whether enrollment occurred before or after the 15th of the calendar month. This approach ensured alignment across patients despite rolling enrollment.

Participants were excluded from the study if they had more than 6 months of inactivity or if they did not complete a baseline DLQI questionnaire within 45 days of enrollment. Participants meeting both exclusion criteria were also excluded from the final analysis.

### Qualitative Data Collection

Patient satisfaction was measured using a feedback form adapted from the Estonian Society of Family Physicians’ standard questionnaire, with detailed information provided in Multimedia Appendix 1. The questionnaire was administered during the final quarter of the pilot project via Google Forms. Responses were collected online, pseudonymized by the research team, and then linked to individual patients in the study database for integration with quantitative data.

### Statistical Analysis

#### Quantitative Data Analysis

##### Descriptive and Comparative Analyses

For both groups, descriptive statistics were used to summarize demographic characteristics and baseline clinical features, including DLQI and PASI scores. The average DLQI change was calculated for each participant using all available follow-up data to account for variability in follow-up completion and differing study start times. This approach allowed the inclusion of participants with incomplete follow-ups while providing a comprehensive measure of QoL improvement. The DLQI change was determined by subtracting the baseline score from the average follow-up score over an 11-month period.

An independent samples *t* test [48] (2-tailed, unpaired) was used to compare mean DLQI change scores between specialist and primary care groups, with the Levene test [49] assessing equality of variances. Treatment effectiveness within the combined care group was evaluated using the Wilcoxon signed rank test [50]. Because of nonnormal data distribution, the Wilcoxon test was applied to the primary care group, while a 1-sample *t* test [51] assessed effectiveness in the specialist care group. The normality of data was verified using the Shapiro-Wilk test [52].

##### Odds Ratios and Correlations

An odds ratio analysis [53] was conducted to compare the likelihood of achieving MCID (DLQI≤–3) between specialist and primary care groups. Additional comparative analyses, including independent samples *t* tests and chi-square tests [54], examined differences between patients who achieved MCID and those who did not. Within the primary care group, Pearson correlation analysis [55] explored associations between DLQI changes and clinical factors such as baseline PASI score, age, sex, disease duration, and use of e-consultation.

##### Selection Bias and Enrollment Timing

To assess potential selection bias introduced by the exclusion criteria, a comparative analysis of baseline characteristics was conducted between included and excluded participants. Variables assessed were age, sex, baseline DLQI, PASI, years since onset, and clinic type (primary care vs specialist care). Independent samples *t* tests and chi-square tests were used to evaluate differences between groups.

Additionally, to explore whether staggered enrollment introduced temporal confounding, a post hoc linear regression model was specified. This model included “enrollment month” as a covariate, alongside clinic type, baseline PASI score, age, and e-consultation frequency. This approach allowed for statistical adjustment of potential timing-related differences in the outcome analysis.

All results with *P* values <.05 were considered statistically significant. Analyses were performed using JAMOVI software (version 2.3.28 [56]; The jamovi project, 2024).

#### Qualitative Case Series Analysis

A case series analysis [57] was conducted on PCP patients who achieved an MCID in the DLQI (≤–3) to explore factors influencing treatment effectiveness at the primary care level. Data sources were reimbursement claims (eg, visits, collaborative care, laboratory tests, diagnoses, and prescriptions), SUS scores, and patient feedback. Although case series analyses carry a higher risk of bias, they are valuable for identifying patterns and building medical knowledge [58]. Patients were selected based on exposure (remote monitoring at the PCP level) and outcome (MCID≤–3), ensuring straightforward and replicable criteria.

Patient-reported experience data were also used to provide additional context for each case. This included SUS scores and a structured feedback form with both closed- and open-ended questions covering onboarding, communication, usability, and satisfaction. While no formal thematic analysis was conducted, selected responses were used descriptively to illustrate variations in patient experience, particularly regarding care communication and perceptions of remote monitoring. Although SUS scores were available for additional participants, analysis was restricted to primary care patients included in the case series who achieved a clinically meaningful DLQI improvement. This approach aimed to provide contextual insights in an exploratory, illustrative manner rather than a comprehensive usability evaluation.

Given the multifactorial influences on QoL—such as treatment type, medication, and collaborative care—remote monitoring has been linked to enhanced patient security [59] and may also be related to anxiety factors [60]. These insights were incorporated to help identify patterns of DLQI improvement and to guide future research.

This manuscript was prepared in accordance with the CONSORT (Consolidated Standards of Reporting Trials)-EHEALTH (version 1.6.1) reporting guidelines, and the completed checklist was submitted as a Multimedia Appendix 2.

### Ethics Approval

The study received ethical approval from the Research Ethics Committee of the University of Tartu, Estonia (ethical approval code 350/T-14). All participants provided written informed consent before enrollment, either in person or electronically, after receiving detailed information about the study and having the opportunity to ask questions.

Data were collected from routine clinical care and study-specific instruments (e.g., questionnaires and photographs). Identifiable data were pseudonymized or anonymized before analysis. Data were securely stored in REDCap (Vanderbilt University; hosted by the University of Tartu) and in a secure teledermatology (TD) platform database, which supported digital patient communication and image handling. Access to both systems was restricted to authorized personnel only.

Pseudonymized data and identification keys will be stored until December 31, 2030, after which the keys will be destroyed. Fully anonymized data will be kept until December 31, 2050, to support future research.

Participation in the study was voluntary, and no compensation was provided to participants.

## Results

### Participant Demographics and Baseline Characteristics

Of the 110 patients enrolled in the intervention, 103 provided DLQI scores. Of these, 76 met the criteria for inclusion in the analysis, with a balanced distribution of males (n=37, 49%) and females (n=39, 51%). The mean age of participants was 43.46 (SD 10.86) years, spanning early to middle adulthood. The mean baseline PASI score was 3.94 (SD 4.46), indicating mild to moderate psoriasis on average. Participants’ baseline DLQI scores averaged 5.28 (SD 4.90), reflecting a moderate impact of the condition on their QoL. Additionally, 27 (36%) participants reported having comorbid conditions, including hypertension (n=8, 30%), dyslipidemias (n=5, 19%), and enteropathic arthropathies (n=4, 15%), among others. The mean duration of psoriasis since onset was 11.70 years (SD 6.12 years), indicating that many participants had lived with the condition for a substantial part of their lives. All results are presented in Table 1.

**Table 1:** Baseline characteristics of included patients.

Characteristics			Combined group (n=76)		Specialist care (n=39)		Primary care (n=37)
**Gender**							
	Sex (male), n (%)	37 (49)		23 (59)		14 (38)	
	Sex (female), n (%)	39 (51)		16 (41)		23 (62)	
Age (years), mean (SD)			43.46 (10.86)		44.33 (12.40)		42.54 (9.04)
PASI^a^, mean (SD)			3.94 (4.46)		3.45 (4.22)		4.47 (4.71)
DLQI^b^ baseline, mean (SD)			5.28 (4.90)		6.21 (5.16)		4.31 (4.49)
Associated diseases^c^, n (%)			27 (36)		13 (33)		14 (38)
Years since onset, mean (SD)			11.70 (6.12)		11.85 (6.60)		11.54 (5.65)

^a^PASI: Psoriasis Area and Severity Index.

^b^DLQI: Dermatology Life Quality Index.

^c^Associated diseases: comorbidities commonly linked with psoriasis such as essential (primary) hypertension, enteropathic arthropathies, disorders of lipoprotein metabolism, overweight and obesity, major depressive disorder, and gout.

A comparison of baseline characteristics between included (n=76) and excluded (n=27) patients was conducted to assess the risk of selection bias resulting from the exclusion criteria. Of the excluded patients, 15 were from primary care and 12 from specialist care.

No statistically significant differences were observed in any of the assessed variables, including age (*P*=.15), sex (*P*>.99), baseline DLQI (*P*=.96), PASI score (*P*=.26), years since onset (*P*=.82), or clinic type (*P*=.54), indicating that exclusions did not systematically bias the final analytic sample. Full comparison results are provided in Multimedia Appendix 3.

### DLQI Changes and Effectiveness Across Care Settings

In the remote monitoring group, DLQI scores decreased by an average of –0.85 (SD 2.89), indicating a general improvement in QoL across the combined care group. The Wilcoxon signed rank test confirmed that this mean change was statistically significant (mean difference –0.85, 95% CI –1.48 to –0.23, *W*=823, *P*=.009).

In the specialist care group, the mean DLQI change was –1.33 (SD 3.12), reflecting a statistically significant improvement (mean difference –1.33, 95% CI –2.34 to –0.32, *t*_38_=–2.66, *P*=.01). By contrast, the primary care group showed a mean DLQI change of –0.34 (SD 2.57), which was not statistically significant (mean difference –0.34, 95% CI –1.00 to 0.42, *W*=259, *P*=.36). Detailed results are presented in Tables 2 and 3.

**Table 2:** Distribution and normality of DLQI^a^ change across care groups.

Group	DLQI change, mean (SD)	Shapiro-Wilk^b^ *P* value	Normality^c^
Remote monitoring group	–0.85 (2.89)	.01	Not normal
Specialist care group	–1.33 (3.12)	.06	Normal
Primary care group	–0.34 (2.57)	.004	Not normal

^a^DLQI: Dermatology Life Quality Index.

^b^Shapiro-Wilk test assesses the normality of distribution.

^c^Normality considered if *P*>.05.

**Table 3:** Statistical test results comparing baseline and follow-up DLQIa scores within care groups.

Group	Test performed	95% CI	Test statistic, *W* or *t* (*df*)	*P* value^b^	Statistical significance^c^
Remote monitoring group	Wilcoxon signed rank test	–1.48 to –0.23	*W*=823	.009	Yes
Specialist care group	One-sample *t* test	–2.34 to –0.32	*t*=–2.66 (38)	.01	Yes
Primary care group	Wilcoxon signed rank test	–1.00 to 0.42	*W*=259	.36	No

^a^DLQI: Dermatology Life Quality Index.

^b^Statistical significance considered if *P*<.05.

^c^Statistical significance “yes” indicates a statistically significant difference; “no” indicates no statistically significant difference. Significance refers to within-group differences in DLQI change scores from baseline, with *P*<.05 considered statistically significant.

In a post hoc linear regression model incorporating enrollment month, clinic type, age, sex, and e-consultation frequency as covariates, no significant association was found between enrollment timing and DLQI change (β=–.025, *P*=.92), indicating that staggered enrollment did not introduce meaningful bias. Full model coefficients are provided in Multimedia Appendix 4.

### Comparison Between Care Types

An independent samples *t* test was conducted to compare the mean DLQI change scores between patients receiving care in specialist settings and those in primary care. The difference was not statistically significant (mean difference 0.99, 95% CI –0.32 to 2.30, *t*_74_=1.51, *P*=.14). The Levene test confirmed the assumption of equal variances (*F*_1,74_=2.12, *P*=.15).

### Odds Ratio and MCID Achievement

The analysis included patients receiving specialist care (n=39) and primary care (n=37). A clinically meaningful improvement in DLQI—defined as a change of ≤–3—was achieved by 10 patients in the specialist care group and 3 patients in the primary care group.

Statistical analysis revealed a significant difference in MCID achievement between care settings (*χ*^2^_1_=4.12, *P*=.04). Patients receiving specialist care were approximately 3.91 times more likely to attain a clinically meaningful improvement in DLQI compared with those in primary care (see Table 4).

**Table 4:** MCID^a^ achievement and odds ratio comparison between specialist and primary care groups.

Metric	Specialist care group (n=39)	Primary care group (n=37)	Combined (n=76)
Total participants, n	39	37	76
Achieved MCID (DLQI≤–3), n (%)	10 (26)	3 (8)	13 (17)
Chi-square test (independence) (*df*), *P* value	4.12 (1), .04	N/A^b^	N/A
Odds ratio (95% CI)	3.91 (0.98-15.57)	N/A	N/A

^a^MCID: minimal clinically important difference.

^b^N/A: not applicable.

### Correlation Analysis in the Primary Care Group

Analysis within the primary care group showed no significant correlation between baseline PASI scores and DLQI change (*r*=0.15, *P*=.38). However, a moderate negative correlation was found between the number of e-consultations—defined here as collaborative care interactions between PCPs and specialists—and DLQI change (*r*=–0.34, *P*=.04). This suggests that increased e-consultations were associated with greater improvements in QoL, reflected by larger decreases in DLQI scores. The key findings are summarized in Table 5.

**Table 5:** Correlation between DLQI^a^ change and clinical variables in the primary care group (Pearson r and 2-tailed *P* value).

Variable			DLQI change		PASI^b^		Sex		Age		Associated diseases^c^		E-consultation		Years since onset
**DLQI change**			—^d^		—		—		—		—		—		—
	*r*	—		—		—		—		—		—		—	
	*P* value	—		—		—		—		—		—		—	
**PASI**			0.147		—		—		—		—		—		—
	*r*	35		—		—		—		—		—		—	
	*P* value	.38		—		—		—		—		—		—	
**Sex**			0.207		0.061		—		—		—		—		—
	*r*	35		35		—		—		—		—		—	
	*P* value	.22		.72		—		—		—		—		—	
**Age**			–0.235		0.039		–0.128		—		—		—		—
	*r*	35		35		35		—		—		—		—	
	*P* value	.16		.82		.45		—		—		—		—	
**Associated diseases**			0.033		0.046		–0.136		–0.006		—		—		—
	*r*	35		35		35		35		—		—		—	
	*P* value	.84		.78		.42		.97		—		—		—	
**e-Consultation**			–0.339		–0.198		–0.184		–0.034		–0.104		—		—
	*r*	35		35		35		35		35		—		—	
	*P* value	*.04* ^e^		.24		.27		.84		.54		—		—	
**Years since onset**			–0.009		0.115		0.036		–0.263		0.171		0.297		—
	*r*	35		35		35		35		35		35		—	
	*P* value	.96		.5		.83		.12		.31		.07		—	

^a^DLQI: Dermatology Life Quality Index.

^b^PASI: Psoriasis Area and Severity Index.

^c^Associated Diseases: comorbidities commonly linked with psoriasis such as essential (primary) hypertension, enteropathic arthropathies, disorders of lipoprotein metabolism, overweight and obesity, major depressive disorder, and gout.

^d^Not applicable.

^e^Statistical significance (*P*<.05).

### Case Series Analysis at the Primary Care Level

#### Overview

Three cases were selected for an in-depth case series analysis to explore potential patterns and gain deeper insights into patient management through remote monitoring at the primary care level.

#### Case 1

A woman in her sixties with mild psoriasis (PASI 0.6) and a baseline DLQI score of 16.0 was enrolled in remote monitoring during the summer. She had been living with the disease for 2 years since its onset.

During the remote monitoring period, the patient’s DLQI score decreased by 6.5 points, indicating a meaningful improvement in QoL. Treatment was coordinated by a large primary care practice comprising 10 PCPs, utilizing a nurse-led management approach. The patient reported high satisfaction with the remote monitoring service and its usability, reflected in an SUS score of 85. She expressed a sense of security provided by the remote care and indicated a preference to continue with this monitoring method. However, she noted that remote monitoring did not reduce her need for in-person health care visits.

The patient reported that she did not receive any active contact from health care providers during the remote monitoring period. No psoriasis-related comorbidities were identified throughout the study. Furthermore, no psoriasis-specific medications were prescribed either before or during the monitoring period, and there were no episodes of collaborative care such as e-consultations with dermatology specialists initiated by the primary care office. Notably, 21 days before the start of remote monitoring, the patient was prescribed methylprednisolone and bilastine to manage allergic reactions.

#### Case 2

A man in his forties with mild psoriasis (PASI 0.2) and a baseline DLQI score of 11.0 points was enrolled in remote monitoring during the spring period. He had been living with the disease for 16 years before enrollment.

During the remote monitoring period, his DLQI score decreased by 8.3 points, indicating a substantial improvement in QoL. The patient was managed in a medium-sized primary care office staffed by 5 PCPs, following a PCP-led care approach. He reported overall satisfaction with the remote monitoring service, assigning an SUS score of 70. He expressed a strong preference to continue with the remote care model and monitoring, highlighting that the remote approach reduced his need for in-person visits and helped him better manage his psoriasis.

The patient also reported being actively contacted by health care providers during the remote monitoring period, which he perceived as supportive. No psoriasis-related comorbidities were detected during the study. Throughout the monitoring period, the patient was prescribed 8 psoriasis-related medications, including calcipotriol + betamethasone and mometasone during the initial months, and methotrexate 4 months after remote monitoring began, following the conclusion of multiple e-consultations with dermatology specialists. The primary care office initiated 4 episodes of collaborative care, including these specialist e-consultations, which appeared to play a role in his treatment management.

#### Case 3

A woman in her forties with mild psoriasis (PASI 0.5) and a baseline DLQI score of 8.0 was enrolled in remote monitoring during the summer period. She had been living with the condition for 4 years at the time of enrollment.

During the remote monitoring period, the patient’s DLQI score decreased by 3.3 points, indicating a moderate improvement in QoL. Treatment was managed by a large 10-PCP office utilizing a nurse-led approach. The patient reported overall satisfaction with the remote monitoring service and its usability, reflected by an SUS score of 72.5. She appreciated clear and sufficient explanations from health care providers, felt well-informed about how to seek help, and valued the opportunity to express her opinions and ask questions regarding her treatment. The patient was satisfied with the speed of both in-person and remote appointments and noted that remote monitoring helped her manage psoriasis better, reducing the need for in-person visits. She expressed a desire to continue using remote monitoring beyond the pilot period.

Notably, the PCP office did not conduct collaborative care episodes, such as e-consultations with specialist dermatologists, during the study period for this patient.

Key patient-level characteristics from the primary care case series are presented in Multimedia Appendix 5.

## Discussion

### Summary of Key Findings

#### Remote Monitoring for Psoriasis: A Mixed Methods Study Across Care Settings

This study used a mixed methods approach to explore the feasibility and effectiveness of remote monitoring for psoriasis across primary and specialist care settings, and to identify factors contributing to better QoL outcomes at the primary care level.

#### Comparative Effectiveness of Remote Psoriasis Management

A comparative analysis measured the effectiveness of remote psoriasis management at both primary and secondary care levels. Consistent with previous studies [4,11], the findings suggest that remote monitoring improves QoL in specialist care settings.

DLQI improvements in the specialist care intervention group (mean difference –1.33, *P*=.01) were comparable to those reported in a similar study [9], which observed a –1.64 change over a 12-month period. Although our study did not use a formal equivalence design and was limited by a smaller sample size, the similarity in effect size supports the clinical relevance of remote monitoring in specialist care settings. These findings should, however, be interpreted with caution and validated in larger studies.

However, improvements in primary care remain limited [20], reflecting systemic challenges such as insufficient training, limited confidence, inadequate resources, and inconsistent guideline adherence among PCPs [18,19,61].

This study extended prior insights from in-person care to remote management, finding that patients in specialist care were 3.91 times more likely to achieve clinically meaningful DLQI improvements than those in primary care. These disparities align with earlier findings [19,20], which show that PCPs are less likely to deliver consistent, guideline-based care due to gaps in knowledge and training [18-20]. Addressing these gaps through standardized guidelines could help ensure consistent care quality across primary and specialist settings, as supported by previous research [62].

#### Collaborative Care Impacts Outcomes at the Primary Care Level

This study also explored factors influencing QoL outcomes at the primary care level. Previous research demonstrated that tools such as e-consultations enhance PCPs’ capacity to manage psoriasis [6,10]. Structured referral protocols with frequent e-consultations have been shown to improve PCP confidence and patient QoL [10], while digital workflows reduce delays and improve outcomes [22].

In this study, PCPs used e-consultations to collaborate with specialists. A correlation analysis found a moderate negative relationship between e-consultation frequency and DLQI change (*r*=–0.339, *P*=.04), supporting the role of frequent specialist involvement in improving outcomes [63,64]. Similarly, the lack of a significant correlation between PASI and DLQI change (*r*=0.142, *P*=.38) in the primary care group aligns with previous research [3,65], suggesting that QoL improvements may depend more on psychosocial support and treatment adherence than on clinical severity. This underscores the need to incorporate patient-centered care models alongside traditional severity metrics. A case-series example illustrated a patient achieving a clinically meaningful DLQI improvement (MCID, defined as a change of ≤–3 points) through multiple collaborative care interventions. The lack of standardized collaborative care protocols in the pilot study may have limited the consistency of these benefits.

#### Variability in Remote Care Management Practices at the Primary Care Level

The case series analysis highlighted variability in remote care management among PCPs, aligning with prior studies that report inconsistent practices in primary care psoriasis management [19,20]. Some patients were managed solely by PCPs, while others were monitored by nurses, reflecting disparities in care approaches. However, the analysis did not identify consistent patterns linking management practices to QoL improvements. These findings reinforce the need for clear, protocol-driven collaborative care models to standardize remote monitoring practices and improve outcomes.

The case series analysis at the primary care level provided additional context to illustrate individual variability in remote psoriasis care and highlight potential mechanisms contributing to QoL improvement. While the sample size was small, these patient-level narratives revealed meaningful differences in care approaches, such as the presence or absence of e-consultations, medication changes, or active patient engagement. This variability helps explain outcome differences that could not be fully captured through quantitative analysis alone. By examining patients who achieved clinically meaningful improvements in DLQI (MCID≤–3), the case series offered insights into how collaborative care, treatment intensity, and user satisfaction may interact to influence outcomes. These illustrative examples support the need for clearer remote care protocols and underscore the importance of personalized care pathways in primary care TD. The findings also highlight the value of mixed methods approaches in uncovering nuanced care dynamics [31,66], and align with previous research showing that patient-reported outcomes are often influenced by psychosocial and engagement factors beyond clinical metrics [3,65].

### Limitations

As a mixed methods study, it balanced quantitative and qualitative data by integrating several complementary research methods, which introduced complexities in design and execution and increased the risk of bias [66].

The relatively small sample size (n=76) limits the generalizability of the findings from the comparative effectiveness and correlation analyses. A larger sample would provide more robust data and enable more definitive conclusions about differences in QoL outcomes between specialist and primary care settings. This study did not perform an a priori sample size calculation, as it was based on data from a real-world pilot intervention [67]. The final sample size was determined by pragmatic and logistical constraints, which may reduce the statistical power of subgroup analyses and the overall generalizability of the findings. Additionally, the study lacked control for confounding factors such as variations in psoriasis severity and treatment adherence.

Although the pilot design did not explicitly control for time-varying external factors, a post hoc analysis incorporating enrollment month found no significant association with DLQI change, indicating limited temporal confounding due to staggered recruitment.

While DLQI improvements were observed, especially in the specialist group, these may partially reflect improved patient adherence or physician-led treatment adjustments in response to remote monitoring data. Although the pilot aimed for patients to complete at least one patient-reported outcome measure each month, several participants submitted DLQI responses only a few times. This limits our ability to assess the long-term effectiveness and sustainability of remote care models based on DLQI data alone. While SUS scores were collected from a broader sample, analysis was limited to the 3 primary care patients included in the case series. Therefore, usability-related findings should be interpreted as illustrative and are not generalizable to the full cohort. Broader usability and access challenges, such as internet connectivity issues, device limitations, or low digital literacy, were not measured but could have influenced patient engagement and outcomes. These factors should be considered in future studies evaluating digital care models.

Although medication and prescription data were available for all participants, a detailed review was conducted only for the 3 primary care patients who achieved an MCID≤–3 in DLQI. This targeted review aimed to explore potential factors associated with clinically meaningful improvement in primary care settings, where overall outcomes were less favorable compared with specialist care. By focusing on patients who demonstrated significant QoL improvement, we sought to identify possible contributors, such as treatment changes, collaborative care episodes, or patient engagement, that may explain better outcomes in the absence of specialist-led management.

These exploratory insights highlighted variability in treatment pathways [30] at the primary care level. However, future research should systematically analyze medication use across the full cohort to better understand the relationship between treatment changes and patient-reported outcomes, and to distinguish these effects from other potential contributors, such as usability, communication, or perceived support.

In addition, comparisons of baseline characteristics between included and excluded patients revealed no statistically significant differences, suggesting a minimal risk of selection bias in the final analytic sample.

The study included a case series analysis to identify factors and patterns. This analysis did not reveal a common pattern contributing to QoL improvement among patients with psoriasis in primary care remote monitoring. However, it also did not contradict the potential factors identified in existing literature and supported by our correlation analysis [57]. The remote monitoring pilot did not prescribe clear protocols for collaborative care or referral practices. Therefore, these factors were investigated using quantitative public claims data alongside less rigorous exploratory methods.

Variability in remote care management approaches among PCPs, coupled with the absence of clear collaborative care protocols, may have limited the consistency and rigor of outcomes observed in this study. Additionally, the study may be subject to selection bias, as participants were recruited from different sites and levels of care. Differences in care delivery models, such as PCP-led versus nurse-led workflows, could have introduced performance bias, potentially affecting the consistency of outcomes.

Based on this, it is clear that any remote monitoring study conducted across different care levels (primary and specialist) should rigorously consider the impact of the health care system’s referral practices on QoL, such as whether general referral, e-consultation, or collaborative care elements are already integrated into the system. These factors should be accounted for, and protocols for using such referral practices should be clearly outlined for study participants.

This is the first study to compare publicly reimbursed remote psoriasis monitoring at both specialist and primary care levels while also evaluating collaborative care tools such as e-consultations and standardized referral protocols aimed at improving patient outcomes. Its limitations provide a foundation for future research, which should address these gaps through well-powered randomized controlled trials that incorporate the factors and insights identified in this study.

### Suggestions for Future Research

Future studies should prioritize developing structured collaborative care models, such as standardized e-consultations, to enhance PCP-led psoriasis management. Another promising research avenue is leveraging public claims data to analyze care processes and identify patterns in referral and treatment behaviors. As noted in prior studies, investigating the psychological effects of remote monitoring could offer valuable insights into how these interventions influence patient security and reassurance.

Moreover, future research should investigate variations in care design and compare the effectiveness of nurse-led, PCP-led, and collaborative approaches. Well-powered randomized controlled trials with larger and more diverse populations are essential to validate findings and enhance our understanding of how remote monitoring can improve QoL outcomes, particularly in primary care settings.

### Conclusions

With limited access to specialists, PCPs play a crucial role in managing psoriasis, underscoring the importance of equipping them with appropriate tools and support. Collaborative practices, such as e-consultations and standardized referral protocols, can enhance care coordination and complement specialist expertise. Providing PCPs with improved resources and guidance can contribute to better patient outcomes, especially in resource-constrained settings.

This study provides unique insights by directly comparing remote monitoring outcomes between specialist and primary care settings. It demonstrates that primary care, when supported by structured resources, can approach specialist-level effectiveness. However, remote management in primary care results in less significant improvements, reflecting systemic challenges also observed in in-person PCP-led care.

These findings highlight the potential of integrating specialist involvement into PCP-led care through standardized e-consultation practices. Leveraging public claims data within a mixed methods approach underscores the need for collaborative care models to ensure consistent quality. Further research should focus on refining and implementing these models to optimize remote psoriasis management in primary care.

## Appendix

Multimedia Appendix 1 Patient satisfaction questionnaire adapted from the Estonian Society of Family Physicians' standard questionnaire.

Multimedia Appendix 2 CONSORT eHEALTH checklist (V 1.6.1)

Multimedia Appendix 3 Exclusion bias analysis.

Multimedia Appendix 4 Regression model Dermatology Life Quality Index (DLQI) change.

Multimedia Appendix 5 Case series summary table.

## Abbreviations

CONSORT: Consolidated Standards of Reporting Trials
DLQI: Dermatology Life Quality Index
MCID: minimal clinically important difference
PASI: Psoriasis Area Severity Index
PCPs: primary care providers
QoL: quality of life
SUS: System Usability Scale
TD: teledermatology

## Data Availability

The data sets generated or analyzed during this study are available from the corresponding author upon reasonable request.
