# Supplementary material for: Remote Monitoring of Psoriasis: Comparing Care Models and Evaluating Quality of Life Outcomes: Mixed Methods Study
Source: J Med Internet Res. 2025 Jun 3;27:e73664. doi: 10.2196/73664 (PMC12174878; doi:10.2196/73664)
Supplement: Multimedia Appendix 1 [file jmir_v27i1e73664_app1.pdf]

**User Experience Feedback Questionnaire**
**Remote Monitoring of Psoriasis**

Dear Patient,

We value the feedback from patients involved in the "Remote Monitoring of Psoriasis" project, aiming to enhance your treatment experience. We would appreciate your input and kindly ask you to answer the following questions regarding your user experience.

**SUS**

Please also respond to the following additional questions.

**FREQUENCY**

How many times have you responded to the psoriasis remote monitoring questionnaires so far?
[1 time, 2-3 times, 4-5 times, 5-10 times, more than 10 times]

Which smart device have you primarily used to respond to the questionnaires?
[Apple phone, Android phone, other phone, Apple tablet, Android tablet, other tablet, regular computer]

**EXPLANATIONS AT THE START OF MONITORING**

Were you informed about how the remote monitoring service would work?
[yes / no]

Did the doctor or nurse take enough time to explain the content of the psoriasis remote monitoring service?
[yes / rather yes / rather no / no]

Were the explanations provided by the doctor or nurse about the remote monitoring service clear?
[yes / rather yes / rather no / no]

Did you feel that you had enough opportunity to express your opinion about the treatment or ask questions when being referred to the service?
[yes / rather yes / rather no / no]

**SMOOTHNESS OF MONITORING**

Have you felt that during the psoriasis remote monitoring service, you knew at any moment whom to ask and how to get help or information?
[yes / rather yes / rather no / no]

Was the information transfer via digital solutions during psoriasis remote monitoring smooth for you?
[yes / rather yes / rather no / no]

If yes, what issues have occurred?

**CONTACT ADDITIONAL TO MONITORING**

Did the doctor or nurse contact you during remote monitoring?
[yes / no]

How often did you receive information from your doctor or nurse about your condition after completing questionnaires and submitting information?
[multiple times a month, once a month, once every two months, once a quarter, once every six months, once a year]

In which way did you communicate with the doctor or nurse about remote monitoring?
[MULTIPLE OPTIONS: only digital solution, phone, video consultation, in-person consultation, other (please specify)]

If you were invited to an in-person consultation, were you satisfied with the speed of getting an appointment?
[yes / rather yes / rather no / no]

If you were invited to a remote consultation (video or phone), were you satisfied with the speed of getting an appointment?
[yes / rather yes / rather no / no]

Would you like to send information to your doctor via the app when the disease flares up?
[yes / rather yes / rather no / no]

How often would you prefer to send information to your doctor or nurse about your condition in this way?
[multiple times a month, once a month, once every two months, once a quarter, once every six months, once a year]

**SATISFACTION**

Did you receive sufficient explanations regarding your health, necessary health exams, or treatment during monitoring?
[yes / rather yes / rather no / no]

Did remote monitoring help you manage psoriasis better?
[yes / rather yes / rather no / no]

Do you think that being in remote monitoring reduced your need for face-to-face doctor visits?
[yes / rather yes / rather no / no]

Were you generally satisfied with the psoriasis remote monitoring service model?
[yes / rather yes / rather no / no]

**CONTINUATION**

Would you like to continue with remote monitoring after the pilot period ends?
[yes / rather yes / rather no / no]

Would you agree if we contact you to ask you to participate in a focus group interview where you can share your opinion on the project and treatment process?
[yes / no]
