# Supplementary material for: Remote Monitoring of Psoriasis: Comparing Care Models and Evaluating Quality of Life Outcomes: Mixed Methods Study
Source: J Med Internet Res. 2025 Jun 3;27:e73664. doi: 10.2196/73664 (PMC12174878; doi:10.2196/73664)
Supplement: Multimedia Appendix 3 [file jmir_v27i1e73664_app3.docx]

**Multimedia Appendix 3. Comparison of Baseline Characteristics Between Included and Excluded Participants**

| **Variable** | **Included (Mean ± SD)** | **Excluded (Mean ± SD)** | **Test (t / χ²)^c^** | **Df^d^** | **p-value^e^** |
| --- | --- | --- | --- | --- | --- |
| Baseline DLQI^a^ | 5.28 ± 4.90 | 5.34 ± 4.55 | t = −0.048 | 93 | 0.962 |
| Baseline PASI^b^ | 3.94 ± 4.46 | 5.43 ± 8.80 | t = −1.123 | 101 | 0.264 |
| Age | 43.5 ± 10.9 | 40.1 ± 9.03 | t = 1.435 | 101 | 0.154 |
| Years since onset | 11.7 ± 6.12 | 11.4 ± 6.54 | t = 0.234 | 101 | 0.815 |
| Sex (1 = F, 2 = M) | 1.49 ± 0.50 | 1.48 ± 0.51 | χ² = 0.000 | 1 | 1 |
| Clinic Type | 37 PC^f^ / 39 SC^g^ | 15 PC / 12 SC | χ² = 0.376 | 1 | 0.54 |

^a^DLQI: Dermatology Life Quality Index.

^b^PASI: Psoriasis Area and Severity Index.

^c^Independent sample t-tests used for continuous variables; chi-square test (χ²) used for categorical variables.

^d^df: Degrees of freedom.

^e^p-value: Statistical significance considered at p < .05.

^f^PC – Primary Care

^g^SC – Specialist care
