# Supplementary material for: Remote Monitoring of Psoriasis: Comparing Care Models and Evaluating Quality of Life Outcomes: Mixed Methods Study
Source: J Med Internet Res. 2025 Jun 3;27:e73664. doi: 10.2196/73664 (PMC12174878; doi:10.2196/73664)
Supplement: Multimedia Appendix 4 [file jmir_v27i1e73664_app4.docx]

**Multimedia Appendix 4.** Linear regression model assessing the association between enrollment month and DLQI change, adjusted for clinic type, age, sex, and e-consultation frequency.

| **Predictor** | **β^a^** | **SE^b^** | **t^c^** | **p value^d^** |
| --- | --- | --- | --- | --- |
| Intercept | 0.752 | 2.379 | 0.316 | .75 |
| Enrolment month | −0.025 | 0.255 | −0.100 | .92 |
| Clinic | −1.262 | 0.791 | −1.596 | .12 |
| Age | 0.023 | 0.031 | 0.744 | .46 |
| Sex | −0.307 | 0.679 | −0.452 | .65 |
| E-consultation | −1.068 | 0.625 | −1.709 | .09 |

^a^β: Unstandardized regression coefficient.

^b^SE: Standard error.

ᶜt: t-statistic from the linear regression model.

^d^p value; values < .05 are considered statistically significant.
