# Supplementary material for: Remote Monitoring of Psoriasis: Comparing Care Models and Evaluating Quality of Life Outcomes: Mixed Methods Study
Source: J Med Internet Res. 2025 Jun 3;27:e73664. doi: 10.2196/73664 (PMC12174878; doi:10.2196/73664)
Supplement: Multimedia Appendix 5 [file jmir_v27i1e73664_app5.docx]

**Multimedia Appendix 5. Summary of Patient-Level Characteristics in the Primary Care Case Series**

| Attribute | Case 1 | Case 2 | Case 3 |
| --- | --- | --- | --- |
| Age Group | 60s | 40s | 40s |
| PASI^a^ | 0.6 | 0.2 | 0.5 |
| Baseline DLQI^b^ | 16 | 11 | 8 |
| DLQI Change | -6.5 | -8.3 | -3.3 |
| PCP Model | Nurse-led, large PCP office | PCP-led, medium PCP office | Nurse-led, large PCP office |
| Active Contact | No | Yes | No |
| SUS Score^c^ | 85 | 70 | 72.5 |
| Medications Prescribed | None (only allergy meds before RM) | Calcipotriol + Betamethasone, Mometasone, Methotrexate | None |
| E-Consultations | None | 4 | None |
| Comorbidities | None | None | None |
| Patient Feedback Summary | Felt secure; RM did not reduce need for in-person visits | Felt empowered; RM reduced in-person visits | Felt informed; RM reduced in-person visits |

^a^PASI: Psoriasis Area and Severity Index.

^b^DLQI: Dermatology Life Quality Index.

^c^SUS: System Usability Scale.
